# Mindset and emotional intelligence in pre-service teachers

**DOI:** 10.1192/j.eurpsy.2024.1247

**Published:** 2024-08-27

**Authors:** M. J. Gutierrez Cobo, R. Cabello, A. Megías-Robles, P. Fernández-Berrocal

**Affiliations:** ^1^Developmental and educational psychology; ^2^Basic psychology, University of Málaga, Málaga, Spain

## Abstract

**Introduction:**

Pre-service teachers must confront emotionally demanding situations associated with the profession, and they must be prepared for it. Previous literature has shown that two variables are important for managing mental health in this population: emotional intelligence (EI) and mindset. EI is the ability to perceive, facilitate, understand, and manage emotions, while mindset refers to beliefs about the malleability of various life domains. According to their mindsets, those who believe that attributes are malleable are called incremental theorists, and those who believe attributes are fixed are entity theorists.

**Objectives:**

This study aimed to explore the influence of intelligence and EI mindset on self-report and ability EI in a sample of 224 female pre-school pre-service teachers (M= 21.27, SD = 4.72).

**Methods:**

Participants completed a questionnaire battery, including intelligence mindset, EI mindset, the Mayer–Salovey–Caruso Emotional Intelligence Test, the Trait Meta-Mood scale, and paternal and maternal educational status.

**Results:**

The results showed that incremental EI theories — but not intelligence — were related to higher scores on self-report and ability EI. Specifically, being an incremental theorist of EI predicted 11% and 20% of the variance in global EI and the managing branch of ability EI, respectively

**Conclusions:**

These results suggest that EI mindset training programs could be implemented and evaluated to explore their impact on the EI of female pre-service teachers

**Disclosure of Interest:**

None Declared

